# Ageism and Health System Responsiveness to Older People: An Agenda for Action and Research

**DOI:** 10.34172/ijhpm.9615

**Published:** 2026-02-14

**Authors:** Thi Vinh Nguyen, Sumit Kane

**Affiliations:** Nossal Institute for Global Health, Melbourne School of Population and Global Health, The University of Melbourne, Melbourne, VIC, Australia.

## Introduction

 Global statistics indicate an increasing trend in the number and proportion of older people within the population, particularly in low- and middle-income countries (LMICs). Estimates suggest that the global population of 60 years and over will nearly double from 12% to 22% between 2015 and 2050, with 80% of older people living in LMICs by 2050.^1^ Of note is that the pace of population ageing in LMICs is higher than high-income countries, meaning that LMICs may have shorter time to prepare for the demographic change. The rapid pace of ageing may widen health gaps and increase inequities and disparities in access to healthcare, not least due to ageing-related multimorbidity burden, poverty, and insufficient preparedness of health systems to address older people’ needs and expectations.

 LMIC governments are aware of these demographic shifts and their implications, and are increasingly responding to these within their policies.^2^ In sub-Saharan Africa, a review found that although many countries have policies on healthy ageing, few have implemented explicit measures to improve older people’s access to existing health services. For example, Ghana, South Africa, and Senegal offer free healthcare or waive health insurance premiums for older people.^2^ In Asia, the Association of Southeast Asian Nations member states have developed national legislation, action plans, and health policies targeting older people.^3^ While these initiatives address important aspects of care, a comprehensive, whole-system approach is required to ensure that older people’s needs and expectations are systematically recognised, prioritised, and addressed across all levels of the health system.

 Health system responsiveness, the ability to respond to people’s non-medical expectations, is one of the three goals of national health systems, alongside good health and fair financial contribution.^4^ Responsiveness refers to how people experience their interactions with the health system along eight domains: dignity, autonomy, confidentiality, prompt attention, access to networks, quality of amenities, choice of providers, coordination, and trust. Both health systems (ie, actors, relations, processes) and the people-related factors (ie, older people, families, communities, their characteristics, needs and expectations) influence these experiences. The larger historical, political, cultural, social, and economic contexts within which health systems and communities are embedded shape people’s interactions and experiences with the health system. Figure presents the conceptual framework for health systems responsiveness to older people; it is adapted from Mirzoev and Kane^4^ and the World Health Organization (WHO).^5^

**Figure:**
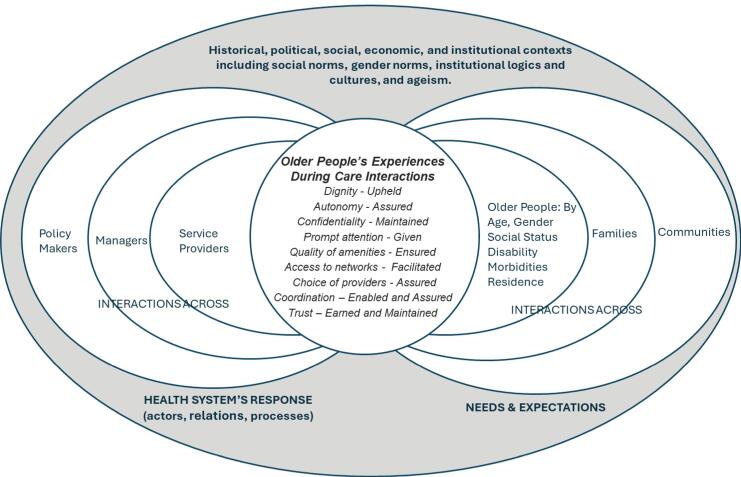
Conceptual Framework for Health Systems Responsiveness to Older People.

 Despite increasing awareness about ageing at the social policy level, ageism remains a significant barrier to equitable healthcare access and outcomes for older people.^6,7^ Ageism is not only linked to older people but can be encountered by other age groups. It encompasses stereotyping, prejudice, and discrimination towards people based on their age.^7,8^ While it can influence any group of age, it predominantly impacts the older population.^7,8^ Ageism can manifest in multiple forms, including institutional, interpersonal, self-directed, or a mix of these types.^6,7^ The 2020 World Values Survey done in 57 countries revealed that ageism was widespread globally; the survey also suggests that people in LMICs are five times more likely to be ageist than in high-income countries.^8^ While systematic assessments of ageism within LMIC health systems are limited, it is not unreasonable to contend that what exists in the society at large would be reflected in health systems too.

 Recognising this, the WHO in its Global Report on Ageism has called for increased research and actions on ageism within health systems in LMICs.^6^ Given the above contexts and in response to this call, this viewpoint highlights specific health system issues rooted in ageism that hinder the capacity to meet the needs and expectations of older people in LMIC contexts. This viewpoint explores ageism through the lens of health system responsiveness and draws attention to how everyday interactions, institutional practices, and system-level functions may shape older people’s experiences of care. This framing complements and extends existing approaches, such as age-friendly services by arguing for a whole-of-system and responsiveness orientation across the WHO health system building blocks across all healthcare settings. The argument being that being age-responsive is everybody’s business, and that policy-makers, managers, providers, and researchers must work together to identify and address ageism, and to enhance health system responsiveness in LMICs. At the midpoint of the Decade of Healthy Ageing, this article serves as a timely call to action for LMICs to prioritise making health systems responsive to the needs and legitimate expectations of older people.

## Ageism in Health Systems in Low- and Middle-Income Countries

 Although the magnitude and effects of ageism on health and well-being of older people are likely to be profound, it has not been prioritised as a public health concern sufficiently yet in LMICs.^8^ Currently, most of research and understanding on ageism towards older people in health systems comes from high-income countries such as Australia, Canada, and the United States, highlighting the need for more and better research and understanding of ageism in LMIC health systems.^9-11^ This lack of comprehensive research on this topic notwithstanding, there is growing awareness of the different aspects of ageism and ageist attitudes within healthcare settings in LMICs.^9^

 Literature from LMICs, though yet limited, reveals significant ageist issues within health systems. Pervasive ageism manifests through the way healthcare providers rationalise, decide, or treat patients differently due to them being older.^6,9,12^ It includes disrespectful and discriminatory attitudes, dismissal of patient’s complaints because of old age, lower treatment priority compared to younger patients, and inadequate provision of information to older people.^9,12^ These patients feel their needs are overlooked, their voice unheard, and they are compelled to be silent and less involved in healthcare decisions than younger people.^12^ A recent example is the evidence that ageist prejudicial practices were common during the COVID-19 pandemic, where the allocation of ventilators and access to intensive care units favored younger people in LMICs, despite older people being more severely affected by the disease.^6^ Such practices are not only ageist, they are unethical and undermine the dignity, autonomy, and trust in patient-provider interactions, and risk older people’s lives. A strong and responsive health system is one that effectively and equitably addresses the legitimate expectations and needs of older patients, their families and communities, yet evidence from LMICs shows systemic failures, leading to a system that inadequately recognises or responds to the diverse needs of older people.

 It is critical to recognise that older people are not a homogeneous group; their needs and expectations vary significantly by age (eg, 60-69 vs. 70-79 years), gender, residence, disability status, dementia, and multiple co-morbidities. These factors intersect, shaping their diverse experiences, capabilities, and needs. However, such variations are often unrecognised and inadequately addressed in LMIC health systems due to entrenched ageism and are often compounded by other forms of inequities such as sexism, ableism, and racism. This occurs through the inadequate attention to and insufficient inclusion of ageing issues in health policies and resource allocation, and low prioritization of the needs and expectations of older people.^13,14^ Many LMICs rely on out-of-pocket payments with insufficient insurance coverage and underfunded health system, disproportionately impacting the poorest older people and those without regular income or health insurance. In India, for instance, 7% of older adults faced catastrophic health expenditures, with out-of-pocket costs particularly high among those with disabilities and low income.^15^ In Malaysia, general practitioners and pharmacists reported that restricted government medication funds constrained effective prescribing, forcing older adults to weigh medication costs before purchase.^16^

 Additionally, poor care coordination and governance within health systems and between health and social services, coupled with inadequate health information systems for analysing and identifying the needs of older people, amplify systemic deficiencies and undermine the care experience of older people.^14^ Evidence from the Philippines and Vietnam showed that integrated, people-centred care for older people continues to be largely informal, ad hoc, and ineffective, with inconsistent collaboration between health and social care workers. This siloed approach results in delayed and insufficient services, duplication of interventions, and significant gaps in care provision and care coordination. At a broader level, a Lancet Global Health commentary highlighted that 75% of LMICs have little or no data to guide planning for older people’s health and social care, leaving policies unaware of the scale of ageing‐related needs.^17^

 Broader entrenched historical, political, cultural, social, and economic contexts shape health system responsiveness to the needs of older people by either challenging or reinforcing ageism.^5^ In LMICs, ageism is largely unchallenged and unrecognised, remaining a neglected social determinant of health and healthcare use.^6^ Some LMIC cultures depict older people as a burdensome, dependent, and less important than younger people, contributing to their neglect and marginalisation within health systems.^9,18^ This cultural portrayal, combined with the prevailing belief that caring for older people is solely the responsibility of their adult children, might explain the continued low attention to older people’s needs in health policies and systems. Older people may internalise these ageist stereotypes and attitudes, known as self-directed ageism, which can shape their perceptions of capabilities, expectations, and needs and influence their health-seeking behaviours, ultimately impacting their healthcare access and outcomes. Addressing these issues requires a shift in recognising that investing in the healthcare of older people is crucial for societal well-being and prosperity.^14^

 While research on ageism in LMIC health systems may be limited, one area that has received scholarly attention relates to the gaps in geriatric training for healthcare professionals and how this may contribute to ageist attitudes and suboptimal care for older people.^9,14^ Available literature suggests that across some LMICs, healthcare professionals often lack training, awareness, skills, and cultural competence to address the specific health and care needs of older people.^9,19^ For instance, a study in Nigeria reported that only 10.8% healthcare workers had learned about ageing during their training, one third knew about specific conditions commonly affecting older people, and only 5% were aware of age-friendly health services.^19^ This evidence implies that insufficient training may lead to a lack of confidence when caringfor older patients, a preference for working with younger patients, and a lower willingness to engage with older patients.^9^ Over time, healthcare professionals, who should ideally advocate for older people, may not recognise ageism, or may inadvertently perpetuate it. This occurs because of both, limited understanding of ageing and ageism, as well as entrenched routines and practices that invisiblise ageism in health systems.^9^ While identifying these gaps in the scholarly literature on medical education is important, it is worth noting that the evidence does not necessarily indicate that developing geriatric speciality services alone is the optimal solution for LMICs. We suggest that a broader, whole-of-system approach, which aims to change the mindset of all providers, all service managers, all health policy actors and to make care responsive to the needs and legitimate expectations of the older people across all care encounters and settings may be more appropriate.

## Enhancing Health System Responsiveness to Older People in Low- and Middle-Income Countries

 In the context of rapid population ageing, there is an urgent need for LMICs to develop health systems that are responsive to the needs of older people. Prioritising action and research on ageism in health systems, we argue, is a necessary step in this direction.^6^ Responsiveness requires that older people’s voices, needs, and expectations are heard, recognised and addressed.^5,20^ It requires that older people are actively involved in decision-making processes related to their own care, and that their inputs to the broader organsiation of care are sought and valued. The point being that health systems can be considered to be strong and responsive not only “when healthcare providers have sufficient skills, autonomy, flexibility and resources to dynamically identify and adapt to the needs and expectations of individual people”^21^ (p. 785), but when the system “as a whole” is adaptive and responsive to the evolving needs of the various population groups it serves.

 We propose an agenda for health systems to tailor their policies, programs, practices and services to meet the needs and legitimate expectations of older people. In line with WHO’s recommendations for aligning health system to deliver integrated care for older adults,^22^ this agenda involves researching and tackling ageism across the six WHO building blocks of health sytems - reframing these as age-responsive service delivery, age-responsive health workforce, age-responsive medical technologies, age-responsive health management information systems (HMIS), age-responsive financing, and age-responsive leadership and governance.The example areas of action and research outlined in Table are drawn from evidence globally, high-income, middle-income, and low-income contexts alike. These areas of action and research need to be contextualised, expanded and specified to country specific needs. They require cooperation between researchers, policy-makers, funders, healthcare managers, healthcare providers, communities, particularly older people and their families, to make health systems age-responsive and to tackle ageism in health systems.

**Table:** Example Areas of Action and Research for Making Health Systems Strong and Responsive to Older People

**Building Blocks**	**Example Areas of Action**	**Example Areas of Research**
Age-responsive leadership and governance	Develop and enforce policies to combat ageism and promote age-responsive health systems^6^ Foster collaboration among policy-makers, healthcare providers, and researchers to implement programs for older people^9^ Strengthen partnerships with private sectors, community organisations, and older people’s unions to improve care and address ageism^14^	Evaluate the effectiveness of current and new anti-ageist and health system responsiveness policies to inform policy refinement, monitoring, and accountability Identify effective leadership and governance for age-responsive health services to guide health system management and reforms^9^ Research on community-based healthcare interventions and model for older people to reduce pressures on health services
Age-responsive service delivery	Develop age-friendly services with geriatric care, comprehensive assessments, and accessible facilities/information^14,23^ Ensure holistic, people-centred care that involves older people and caregivers in decision-making, beyond family responsibility Provide regular community- and home-based healthcare services for older people	Conduct research on expectations and needs of older people about age-responsive health systems to inform service design and quality improvement^14^ Investigate prevalence, forms, drivers, and impacts of ageism in health systems, using WHO’s newly launched ageism scale to enable cross-setting comparison^24^ Conduct implementation research to evaluate effectiveness of different interventional models in addressing ageism in health services^6^
Age-responsive health workforce	Integrate care for older people, ageism awareness, and intergenerational learning into medical and allied health curricula^6^ Provide ongoing training, mentorship, and capacity-building for healthcare and non-health staff, managers, and policy-makers in care for older people^23^ Facilitate knowledge exchange between high-income countries and LMICs to adapt best practices and advanced models of care	Examine healthcare professionals’ experiences, needs, and challenges regarding ageism and care for older people to guide curriculum development and continuing education Assess the impact of ageism training on staff attitudes, practices, and patient-reported responsiveness Identify effective training approaches on ageism and ageing through implementation research
Age-responsive technologies	Safely include older people in research to ensure treatments and vaccines are effective and safe Improve older people’s access to medical treatments, therapies, vaccines, and assistive technologies, with regular accessibility reviews Offer mHealth, telehealth, and virtual or remote health check-ups for older people	Identify barriers to accessing medical products, assistive technologies, vaccines for older people Research effective models to provide mHealth and telehealth for older people to address digital barriers and improve access to care for older people
Age-responsive HMIS	Establish mechanisms to collect feedback from older patients and caregivers and use it to improve care Collect and analyse data on ageism through HMIS and national health or ageing surveys^6^ Establish data linkage between health systems and with other social systems for older people to better identify and address their needs	Assess the effectiveness of using routine data from HMIS in identify and address ageism and healthcare needs of older people Identify methods to use age-integrated data in HMIS for policy- making, resource allocating, and services planning
Age-responsive financing	Provide adequate budget allocation for health programs and services to older people Develop health insurance schemes that cover comprehensive healthcare for older people	Evaluate the coverage and impact of health insurance schemes on older people’s health outcomes to support universal health coverage for ageing populations Evaluate the cost–effectiveness of different intervention models and new policies to guide resource allocation and inform policy and programs

Abbreviations: LMICs, low- and middle-income countries; HMIS, health management information systems; WHO, World Health Organization.

## Conclusion

 In this article, we make a case for age-responsive health systems and specifcially argue that ageism needs to be actively identified and tackled within health systems. We contend that not doing so not only hinders the well-being of older people, it damages provider-patient relationships and trust across society, and negatively affects healthcare access and outcomes for all. We assert that to address ageism and to make health systems age-responsive requires comprehensive research and action across all aspects of the health system, and needs active involvement of healthcare providers, manager, policy-makers, and of older people, their families, and communities. We propose an agenda for action and research towards making health systems age-responsive, strong, and resilient.

## Disclosure of artificial intelligence (AI) use

 Not applicable.

## Ethical issues

 Not applicable.

## Conflicts of interest

 Authors declare that they have no conflicts of interest.

## References

[1] World Health Organization. Ageing and health 2022. https://www.who.int/news-room/fact-sheets/detail/ageing-and-health.

[2] Saka S, Oosthuizen F, Nlooto M (2019). National Policies and Older People’s Healthcare in Sub-Saharan Africa: A Scoping Review. Ann Glob Health.

[3] ASEAN Secretariat. Policy developments on population ageing and empowering older persons in ASEAN member states 2019. https://www.unescap.org/sites/default/files/Policy%20Developments%20on%20Population%20Ageing%20by%20ASEAN%20Secretariat.pdf. Accessed September 5, 2025.

[4] World Health Organization (WHO). The World Health Report 2000: Health Systems: Improving Performance. WHO; 2000.

[5] Mirzoev T, Kane S (2017). What is health systems responsiveness? Review of existing knowledge and proposed conceptual framework. BMJ Global Health.

[6] World Health Organization (WHO). Global report on ageism. Geneva: WHO; 2021.

[7] Wyman MF, Shiovitz-Ezra S, Bengel J. Ageism in the health care system: providers, patients, and systems. In: Ayalon L, Tesch-Römer C, eds. Contemporary Perspectives on Ageism. Cham: Springer International Publishing; 2018. p. 193-212.

[8] Officer A, Thiyagarajan JA, Schneiders ML, Nash P, de la Fuente-Núñez V (2020). Ageism, healthy life expectancy and population ageing: how are they related?. Int J Environ Res Public Health.

[9] Ugargol A, D’Souza P. Addressing Ageism in Healthcare: Insights for an Age-Inclusive Longevity Society. Springer; 2024. p. 1-24.

[10] North MS, Fiske ST (2015). Modern attitudes toward older adults in the aging world: a cross-cultural meta-analysis. Psychol Bull.

[11] Ayalon L, Dolberg P, Mikulionienė S, Perek-Białas J, Rapolienė G, Stypinska J (2019). A systematic review of existing ageism scales. Ageing Res Rev.

[12] Motsohi T, Namane M, Anele AC, Abbas M, Kalula SZ (2020). Older persons’ experience with health care at two primary level clinics in Cape Town, South Africa: a qualitative assessment. BJGP Open.

[13] Burnes D, Sheppard C, Henderson CR Jr (2019). Interventions to reduce ageism against older adults: a systematic review and meta-analysis. Am J Public Health.

[14] Goharinezhad S, Maleki M, Baradaran HR, Ravaghi H (2016). A qualitative study of the current situation of elderly care in Iran: what can we do for the future?. Glob Health Action.

[15] Brinda EM, Kowal P, Attermann J, Enemark U (2015). Health service use, out-of-pocket payments and catastrophic health expenditure among older people in India: the WHO Study on global AGEing and adult health (SAGE). J Epidemiol Community Health.

[16] Christopher CM, Loong MCW, Blebil AQ (2023). Helping older adults with their medication use problems: A qualitative study on perspectives and challenges of primary health care providers. Arch GerontolGeriatr.

[17] Goodman-Palmer D, Ferriolli E, Gordon AL (2023). Health and wellbeing of older people in LMICs: a call for research-informed decision making. Lancet Glob Health.

[18] Araújo PO, Soares I, Vale P, Sousa AR, Aparicio EC, Carvalho ESS (2023). Ageism directed to older adults in health services: a scoping review. Rev Lat Am Enfermagem.

[19] Ogunyemi AO, Balogun MR, Ojo AE (2023). Provider and facility readiness for age-friendly health services for older adults in primary health care centres in southwest, Nigeria. PLOS Glob Public Health.

[20] Nguyen TV, Kane S (2024). Towards an agenda of action and research for making health systems responsive to the needs of people with disabilities. Lancet Reg Health West Pac.

[21] Bridges J, Pope C, Braithwaite J (2019). Making health care responsive to the needs of older people. Age Ageing.

[22] World Health Organization (WHO). Integrated care for older people: Realigning primary health care to respond to population ageing. WHO; 2018.

[23] Karami B, Ostad-Taghizadeh A, Rashidian A, Tajvar M (2023). Developing a conceptual framework for an age-friendly health system: a scoping review. Int J Health Policy Manag.

[24] World Health Organization (WHO). WHO Ageism Scale: Manual and User Guide. Geneva, Switzerland: WHO; 2025. https://www.aworld4allages.org/who-ageism-scale.

